# Clinical Features and Treatment Strategies of Li‐Fraumeni Syndrome Patients With Inherited TP53 Mutations

**DOI:** 10.1002/mgg3.70177

**Published:** 2026-01-07

**Authors:** Keyu Chen, Yufen Xu, Binbin Song, Yuyang Gu, Xiaofang Xu, Jun Cao, Meiyu Fang

**Affiliations:** ^1^ The First Hospital of Jiaxing & The Affiliated Hospital of Jiaxing University Jiaxing China; ^2^ Key Laboratory of Head & Neck Cancer Translational Research of Zhejiang Province, Department of Head and Neck and Rare Oncology, Zhejiang Cancer Hospital, Hangzhou Institute of Medicine (HIM) Chinese Academy of Sciences Hangzhou China

**Keywords:** germline mutations, hereditary disease, immune checkpoint inhibitors, Li‐Fraumeni syndrome, *TP53* pathogenic/likely pathogenic variants

## Abstract

**Background:**

Li‐Fraumeni syndrome is a rare autosomal dominant disorder caused by a pathogenic mutation of the tumor suppressor gene *TP53*. This disease starts at an early age and has been shown to be associated with multiple tumors. The study aims to discuss the clinical and genetic characteristics of Li‐Fraumeni syndrome (LFS) and to provide therapeutic experience of LFS.

**Materials and Methods:**

We conducted a retrospective analysis of the clinicopathologic features, family history, treatment and follow‐up in five LFS patients with germline *TP53* (NCBI Gene: 7157, HGNC: 11998, OMIM: 191170) pathogenic/likely pathogenic (P/LP) variants. This research had been approved by the ethics committee and implemented.

**Results:**

Our study involved five LFS patients with germline *TP53* P/LP variants, including thyroid cancer, ovarian melanoma, colon cancer, fibrosarcoma, and lung cancer. Among this group of patients, the age at which tumors first appeared was between 24 and 53 years old. Three patients had a family history of tumors, and the other two were probands in the family. Traditional chemotherapy has limited effectiveness in clinical practice and may increase the risk of tumor development. However, immune checkpoint inhibitors (ICIs) have shown unexpected efficacy in patients with high programmed cell death ligand‐1 (PD‐L1) expression. Next‐generation sequencing (NGS) and PD‐L1 detection may provide more potential targets for LFS patients to achieve better therapeutic outcomes. In addition, we have added a new *TP53* frameshift mutation spectrum, namely c.642_643delTA (p.H214Qfs*7), which belongs to the pathogenic variant. This mutant has not been described in the existing literature.

**Conclusion:**

Patients with LFS may be potential beneficiaries of immune checkpoint inhibitors and targeted therapies.

## Graphical abstract

LFS with TP53 disease‐causing mutations may be potential beneficiaries of immune checkpoint inhibitors and targeted therapies. Therefore, NGS and PD‐L1 testing should be performed in all LFS patients.

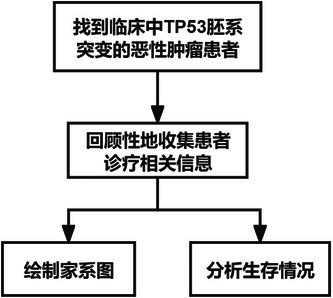

## 1 Introduction

*TP53* (*191170) is the most commonly mutated gene in human cancers and coordinates the cellular response to DNA damage (Liu et al. 2019). Mutations in this gene often lead to a loss of activity in the protein that codes for *p53* (Liu et al. 2019). Somatic mutations in the *TP53* gene are present in more than 50% of tumors (Liu et al. 2019). Germline *TP53* pathogenic/likely pathogenic (P/LP) variants are the basis for diagnosis of Li‐Fraumeni syndrome (LFS). According to the studies in the United States and European, the frequency of germline *TP53* mutations is about 0.02% in the population, which tends to lead to early‐onset of cancer (Lalloo et al. 2003). 50% of patients are found to have malignant tumors before the age of 30. Here, we report five patients with germline *TP53* pathogenic variants. Two of them have thyroid cancer, one has malignant melanoma, and the other two have two primary tumors.

## 2 Clinical History

### 2.1 Case 1

A young man was diagnosed with follicular thyroid carcinoma at the age of 24 years, with multiple cervical lymph nodes and bilateral lung metastases. Due to compression, stenosis, and displacement of the trachea, he underwent total thyroidectomy and deep lymphadenectomy. It was 5.5 × 4 × 3.5 cm. The histopathology considered it to be follicular thyroid carcinoma, consistent with a poorly differentiated type. One month after surgery, the patient developed multiple lymph node enlargement in the lower right neck and was subsequently treated with radioiodine. Only 4 months later, the patient presented with multiple enlarged lymph nodes in the neck again, including supraclavicular lymph nodes. The larger one was located in the lower right neck, with dimensions of 35 mm × 15 mm. At the same time, the lung lesions also gradually enlarged and increased. In palliative resection of the neck, it was found that the tumor had invaded the thyroid cartilage, cricoid cartilage and even infiltrated the laryngeal cavity, so radical resection was not performed. Considering the high degree of malignancy, strong aggressiveness and rapid progression of the tumor, doxorubicin liposomes and cisplatin were administered for 6 cycles starting from 3 weeks after surgery. During treatment, the patient's condition was stable, the best response to pulmonary lesions was stable disease (SD), and the cervical lymph nodes achieved partial response (PR). However, less than a month after the chemotherapy ended, lesions in both lungs and neck appeared to progress. The biopsy pathology of lung lesions first considered metastasis of thyroid cancer. Tumor cells were diffusively positive for TTF‐1, thyroglobulin (TG) and CK7. During this period, we detected the presence of *TP53* pathogenic variants (frameshift mutation, c.642_643delTA, p.H214Qfs*7) in the tumor tissue. Subsequently, he received treatment with anti‐angiogenic drugs and programmed cell death protein‐1 (PD‐1) monoclonal antibody, including anlotinib hydrochloride capsules and toripalimab injection. Although his lesions remained relatively stable during treatment, at the 8 months of medication, he developed severe pneumothorax in his left lung, which compressed the lung by nearly 70%. The lung lesions were still stable. Three months after stopping treatment, his disease also progressed. Finally, he died of tumor‐related complications, with an overall survival (OS) of 37 months.

Prior to his diagnosis, there was no history of malignant tumors in his family.

### 2.2 Case 2

A 35‐year‐old woman was further examined following the discovery of a mass on her right neck. PET/CT suggested thyroid malignancy with bilateral lungs, sternal, and mediastinal lymph node metastasis. The patient subsequently underwent total thyroidectomy, as well as cervical and mediastinal lymph node dissection. The histopathology indicated that the tissue tended to undifferentiated thyroid, with a high possibility of transformation from low‐differentiated to undifferentiated carcinoma. The tumor had a maximum diameter of 4.5 cm and invaded the capsule. Immunohistochemical (IHC) analysis showed that TTF‐1 and PAX8 staining were positive, and CK7 was partially positive. Surprisingly, TG was negative. Through tumor multigene assay by next‐generation sequencing (NGS), we found germline *TP53* pathogenic variants (frameshift mutation, c.642_643delTA, p.H214Qfs*7). In addition, the combined positive score (CPS) for PD‐L1 expression was 70%. Therefore, the patient underwent a strategy of AP regimen, including chemotherapy (doxorubicin liposome + cisplatin) and immunotherapy (pembrolizumab). During this period, cisplatin was discontinued due to abnormal renal function, but regular review indicated that the disease was stable. At present, the patient is still on immune monotherapy. She has no disease progression for 3 years after diagnosis.

Notably, she had a significant family history of malignancies, including breast cancer, lymphoma, lung cancer, etc. In this family, her father had multiple primary tumors, including gastric, colon, lung, and renal cancer, while her brother was diagnosed with lymphoma at the age of 35. All of the affected family members had cancer detected before the age of 50, with the youngest family member diagnosed at age 22.

### 2.3 Case 3

A 31‐year‐old woman underwent surgery for pelvic tumors and was pathologically diagnosed as malignant melanoma of the ovary. The tumor cells were strongly positive for Vim, MelanA, HMB45, S‐100, CD56, and SOX10. Immunohistochemistry revealed that mutations of *p53* were detected in tumor cells. On further examination, we detected a *TP53* pathogenic variant (missense mutation, c.742C>T, p.R248W). PET/CT examination was completed after surgery, and no other suspicious lesions were found. She was then treated with pembrolizumab. But just 2 months after surgery, she had a recurrence of the disease, including multiple lesions in the pelvic cavity, abdominal wall, and groin. She underwent extensive resection and lymph node dissection again. The postoperative pathology was still considered malignant melanoma. After the second surgery, she received radiotherapy to the left pelvic wall and inguinal area, along with the antitumor therapy of lenvatinib and pembrolizumab. About 9 months after treatment, she developed abdominal implantation metastasis with malignant ascites. Since then, she began to receive chemotherapy, targeted therapy, and immunotherapy (albumin‐bound paclitaxel + cisplatin + lenvatinib + pembrolizumab). During the period, there was a temporary improvement, but the disease still progressed 4 months later. Later, she developed uncontrolled hydrops abdominis, and unfortunately died. From diagnosis to death, the OS was just 18 months.

Before being diagnosed with malignant melanoma of the ovary, she had suffered from a history of multiple malignancies, including fibrous histiocytoma, breast cancer, and hepatic precancerous lesion. In her maternal family, her mother had breast cancer and ovarian teratoma, while her grandfather and maternal uncle both had lung cancer.

### 2.4 Case 4

A male patient was diagnosed with moderately differentiated adenocarcinoma of the ascending colon at the age of 33. He subsequently underwent right hemicolectomy and conventional chemotherapy (oxaliplatin + capecitabine). After 12 years, he was diagnosed with a second primary tumor, which was pathologically considered to be myxofibrosarcoma. *P53* was positive in tumor cells. High‐throughput sequencing results of sacrococcygeal excision tissue (namely the second primary tumor) showed that it had germline *TP53* variants (R110L, heterozygous mutation). After surgery, he received 6 cycles of chemotherapy (ifosfamide + doxorubicin liposome) and adjuvant radiotherapy of sacrococcygeal tumor bed. Two years later, the patient was still alive and there was no progression of the tumor.

In his family, there were no other cancer patients.

### 2.5 Case 5

A 53‐year‐old woman inadvertently discovered a mass in her lower back that was progressively enlarged. CT showed a left ventral and dorsal soft tissue mass with a diameter of 7.8 cm, involving the intercostal space. Furthermore, there were patchy, dense shadows in the upper lobe of her left lung. The biopsy pathology of the back mass firstly considered sarcoma. She then underwent radical surgery for lung cancer and extensive excision of tissue tumors. Postoperative pathology confirmed that the lung lesion belonged to lung adenocarcinoma, while the dorsal tumor was consistent with myxofibrosarcoma. The lung lesion was an early‐stage tumor (Ia), and chemotherapy was not required. Therefore, she received 5 cycles of adjuvant chemotherapy (ifosfamide + doxorubicin) and radiation therapy for soft tissue sarcoma. About 3 years after surgery, the mass reappeared on her left back. The biopsy pathology considered tumor recurrence. Therefore, she received targeted therapy (anlotinib, 12 mg oral) and PD‐1 immunotherapy. Over the next year, the left shoulder mass still recurred repeatedly after surgical removal and debridement. Therefore, the second‐line treatment was changed to gemcitabine combined with dacarbazine. During this period, CT examination indicated that the lesion continued to grow slowly until brain metastases appeared. In the next 6 months, she received a variety of treatment regimens, such as albumin‐bound paclitaxel combined with gemcitabine, cranial radiotherapy, doxorubicin liposome combined with lenvatinib, and ifosfamide combined with lenvatinib, but the response to chemotherapy was not satisfactory. She was still alive 8 months after the diagnosis of brain metastases.

In the patient's first recurrence of dorsal mass, germline *TP53* likely pathogenic variants were detected in the tissue (*TP53* missense mutation, c.644G>A, p.Ser215Asn, heterozygous mutation). In her family, both her mother and elder brother had been diagnosed with lung cancer, and her mother died of this disease.

## 3 Results

We report 5 patients with germline *TP53* P/LP variants, including three females and two males. The clinical, genetic and epigenetic characteristics of the cohort are summarized in Table 1. They were first diagnosed with malignancy between the ages of 24 and 53, and 80% (4/5) were diagnosed with malignancy before the age of 35. Two of five patients had poorly differentiated or undifferentiated thyroid cancer; one of them had malignant melanoma of the ovary, and the other two had a history of colon cancer (case 4) or bilateral lung adenocarcinoma (case 5) in addition to myxofibrosarcoma.

**TABLE 1:** clinical, genetic and epigenetic characteristics.

Patients	Case 1	Case 2	Case 3	Case 4	Case 5
Age (years)	24 years	35 years	31 years	33 years	53 years
Sex	Male	Female	Female	Male	Female
Family history	No	Yes	Yes	No	Yes
Primary tumor	Thyroid cancer	Thyroid cancer	Malignant melanoma of the ovary	Colon cancer (33 years) Sacrococcygeal mesenchymal tumor (45 years)	Bilateral lung cancer Soft tissue sarcoma
Pathological type	Poorly differentiated carcinoma	Undifferentiated carcinoma	Malignant melanoma	Moderately differentiated adenocarcinoma Myxofibrosarcoma	Adenocarcinoma Myxofibrosarcoma
Metastatic site	Lung and lymph node	Lung, bone and lymph node	Parietal and parietal tubercles, inguinal lymph nodes and peritoneum	None	Local recurrence, pleural and bone metastases
*TP53* germline variant	c.642_643delTA p.H214Qfs	c.642_643delTA p.H214Qfs	c.742C>T (p.R248W)	R110L	c.644G>A (p.Ser215Asn)
Status (overall survival)	Dead (37 months)	Alive	Dead (18 months)	Alive	Alive

Of these patients, only one patient (case 4) had no local recurrence or distant metastasis 2 years after the discovery of the second primary tumor, while all the other patients had recurrent recurrence or significant organ metastasis.

## 4 Discussion

Li‐Fraumeni syndrome (LFS) is a rare autosomal dominant genetic disorder caused by germline *TP53* pathogenic/likely pathogenic (P/LP) variants (Schneider et al. 1993; Malkin 2011). This disease was first described by Frederick Li and Joseph Fraumeni in 1969 while studying childhood and familial cancers (Li and Fraumeni Jr. 1969a, 1969b). The frequency of diverse cancer types observed in such families far exceeded the sporadic level, and the risk of cancer was very high in childhood (de Andra et al. 2021). Compared with the general population, the incidence of any cancer was nearly 24 times higher in individuals with LFS, with the highest relative incidence from childhood to adolescence (de Andra et al. 2021). According to statistics, about half of LFS‐related malignancies occurred before 30 years old (Chompret et al. 2000; Li et al. 1988). In males with LFS, the lifetime risk of cancer is 73%, while it is nearly 100% in females. Typical tumor types include early‐onset bone and soft tissue sarcomas, breast cancer, adrenocortical carcinoma, central nervous system tumors, and acute leukemia, which are known as the “core” cancers of LFS (Li et al. 1988; Bougeard et al. 2015). Other less common tumors, such as thyroid cancer, prostate cancer, lung cancer, and colon cancer, may also be associated with LFS. Many patients with LFS may develop more than one primary cancer during their lifetime. Among the patients we included, the earliest onset was only 24 years old. The patient died of the disease progression within 3 years after the onset of the disease.

There are many different criteria used to identify individuals with a high likelihood of LFS. To be diagnosed as a classic LFS family, three criteria must be met. First, the proband was diagnosed with sarcoma before age 45; second, first‐degree relatives, including parents, siblings, and children, developed any cancer before age 45; finally, another first‐ or second‐degree relative developed any cancer before age 45, or had sarcoma at any age (Li et al. 1988). The Chompret criteria further expand the scope of disease by meeting any of the following criteria: (1) The proband had “core” tumors belonging to LFS tumor spectrum before age 46 and at least a first‐ or second‐degree relative with a LFS‐related tumor (if the proband had breast cancer, breast cancer should be excluded) before age 56, or the proband suffered from multiple types of tumors; (2) The proband had multiple tumors (excluding multiple breast tumors), among which two cases belonged to the LFS tumor spectrum and the first tumor was before the age of 46; (3) Patients with rare tumors such as adrenocortical carcinoma, choroid plexus, or embryonal anaplastic rhabdomyosarcoma; (4) Patients who were diagnosed with breast cancer before the age of 31 (Bougeard et al. 2015). The latter is one of the most widely used diagnostic criteria. Among the five patients involved in this paper, the families of Case 2, Case 3, and Case 5 all had tumor history of close relatives. In Case 2 families, the patient was diagnosed with undifferentiated thyroid cancer at the age of 35. Several first‐ and second‐degree relatives in her family all had a history of malignant tumors, and the age of onset of the primary tumors was all less than 50 years old. Among them, the patient with the earliest age of onset was only 22 years old. In Case 3 families, the patient had a history of breast cancer before age 31 and subsequently developed malignant melanoma of the ovary, which met the Chompret criteria for early‐onset breast cancer. Case 5 had multiple tumors, including a soft tissue sarcoma that belonged to the LFS tumor spectrum. The classical criteria and Chompret criteria for the diagnosis of LFS are mainly based on the clinical characteristics of the patient (disease, age of onset) and familial pathogenic characteristics (family history of malignancy). Due to the influence of family planning policy, many Chinese families have no siblings. For example, the families of Case 1 and Case 4 had no other members with a history of tumors. For families without an available family history, it is difficult to assess risk using conventional criteria. Of course, it is also possible that acquired factors caused de novo mutations in the genes of the two probands. Although the frequency of this phenomenon has not been confirmed, studies by Gonzalez have indicated that the incidence may fluctuate between 7% and 20% (Gonzalez et al. 2009).

Furthermore, with the development of genetic testing technology, genes associated with hereditary tumors are gradually identified. *TP53* is a tumor suppressor gene located on chromosome 17p13.1. It produces the protein *p53* by transcription, thereby delaying the cell cycle and providing an opportunity for cell repair. Once *TP53* mutates, damaged DNA inside the cell may become cancerous because it cannot be repaired (Liu et al. 2019; Zhu et al. 2024). Numerous studies have shown that approximately 70% of typical LFS families are attributed to germline *TP53* P/LP variants (mutations) (Li et al. 1988; Bougeard et al. 2015; Frebourg et al. 1995, 2020; Birch et al. 1994). This is the only confirmed potential molecular basis for the diagnosis of LFS. Normally, the cell carries two copies of the *TP53* gene, one from the father and the other from the mother. If both copies of the *TP53* gene are damaged (inherited from a single parent with the same disease or a new *TP53* mutation), cancer may develop (Birch et al. 1994). Among patients with germline pathogenic variants in *TP53*, the cumulative cancer incidence is almost 100% at age 70, regardless of gender (Mai et al. 2016). The large‐sample study by de Andrade had indicated that the prevalence of *TP53* potentially pathogenic variants in the general population may be up to 10 times higher than the estimated results based on family studies (de Andra et al. 2017, 2019). In certain cancer‐susceptible populations, they appeared a similar pattern of onset as patients with LFS. However, no pathogenic mutation of *TP53* was detected. It maybe related to the involvement of MDM2 and CHEK2 genes (Malkin 2011). Among the five cases of LFS we explored, the NGS indicated the presence of germline *TP53* P/LP variants, but only three cases had a family history of malignancy, while the other two were progenitors in the family. Among them, Case 2 and Case 3 had been first reported on Research Square by Tian from the perspective of molecular diagnosis (Tian et al. 2024). It should be pointed out that the article is currently in preprint. In the cases we reported, we present two cases associated with a new frameshift mutation, which is *TP53* c.642_643delTA (p.H214Qfs*7) mutant spectrum. Both of these two patients suffered from poorly differentiated thyroid carcinoma. This mutation of *TP53* indicates that the reading frame shifts from amino acid 214 and terminates 7 downstream residues, resulting in a premature truncation of the 393 amino acid *TP53* protein and a loss of *TP53* protein function. The ClinVar database was first recorded in May 2020 and noted that this mutation was a pathogenic mutation. Despite receiving aggressive antitumor therapy, Case 1 still died of rapid tumor progression and severe complications, while Case 2 still benefited from current treatment strategies (OS more than 3 years). Since cases of this mutant have never been reported, we cannot make a prognosis for this type of mutation at this time. The missense mutation, *TP53* p.R248W, is one of the common hot spot mutations. In preclinical studies, this mutant exert gain‐of‐functions through constitutively binding to and hyperactivating STAT3, leading to increased proliferation and aggressiveness of tumor cells (Klemke et al. 2021). This is often associated with a poorer prognosis. Case 3 is also a mutation at this site. Only 2 months after surgery, the patient experienced a recurrence of the disease. Despite receiving aggressive antitumor therapy, her overall survival was only 1.5 years. The *TP53* R110L is a relatively rare mutation and has not been widely reported in the literature. In the AACR Project GENIE case, this group is present for only 0.10% (AACR Project GENIE Consortium 2017). Compared to other types, this germline mutation appears to have a relatively good prognosis. Case 4 was still able to achieve a cured state and survive for a long time after experiencing two types of malignancies. The *TP53* c.644G>A (p.Ser215Asn) variant is a likely pathogenic locus associated with inherited cancer susceptibility syndromes. Although this variant has been mentioned in a variety of tumor species, including breast cancer, soft tissue sarcoma, lung cancer, and thyroid cancer, we did not find a correlation between this variant and clinical prognosis. From the five patients we collected, it can be seen that different *TP53* variants may have different prognoses, but it cannot be ruled out that the primary disease itself has a poor prognosis.

In modern clinical practice, surgery, chemotherapy, and radiotherapy are the most important methods for the treatment of cancer. But previous studies have shown that the expression of germline *TP53* mutation makes cells more resistant to radiation by affecting their response to deoxyribonucleic acid (DNA) damage (Lee and Bernstein 1993; Matsui et al. 2001). In addition, a higher rate of secondary primary tumors in the radiation field following the presence of ionizing radiation has also been described in LFS patients. According to the Hendrickson study, patients have an estimated 30% increased risk of secondary malignancy from LFS associated radiation (Le et al. 2020). However, radiation therapy is not an absolute contraindication for patients with LFS. Multiple clinical data have suggested that patients with LFS have a lower risk of radiation‐induced secondary malignancies than previously reported in the literate (Le et al. 2020; Hendrickson et al. 2020; Petry et al. 2020). Therefore, when choosing adjuvant radiotherapy, oncologists should first weigh the long‐term risk of radiation‐induced cancer associated with LFS against the potential risk of local recurrence without radiotherapy. In addition to radiation exposure, animal studies have manifested that some conventional chemotherapy, such as the topoisomerase inhibitor etoposide, significantly increase the risk of tumor development in mouse models with LFS. However, clinical evidence for this phenomenon is still lacking (Kasper et al. 2018). To reduce the risk of multiple primary tumors in patients with germline *TP53* pathogenic mutations, clinicians should avoid cytotoxic drug therapy whenever possible and give priority to surgical treatment. From another point of view, in the case that surgery cannot eradicate the mass or the disease recurs repeatedly affecting the patient's life and mental health, conventional chemoradiotherapy is an option.

Besides surgery and conventional chemoradiotherapy, targeted therapy and immunotherapy may be candidates for patients with LFS (Light et al. 2023). In a prospective cohort study, Light identified clinically accessible site mutations, such as BRAF V600E mutations, NF1 mutations, and hypermutation indicating the efficacy of immune checkpoint inhibitors, in three pediatric cancer patients with germline *TP53* pathogenic mutations (Light et al. 2023). Research by Mezquita revealed that 90% of LFS patients with lung adenocarcinoma had driver gene mutations, mostly EGFR mutations, and the frequency of EGFR mutations was higher than that of the general lung adenocarcinoma population (Mezquita et al. 2020). Among the patients included in our study, except for case 4, who only received traditional surgical treatment and chemoradiotherapy, all other patients had a record of targeted and/or immunotherapy and benefited to some extent. It was noteworthy that PD‐L1 expression in Case 2 was as high as 70%, and there was no disease progression after nearly 3 years of immunotherapy. Although there are no large clinical studies evaluating the efficacy of targeted and immunotherapy in patients with LFS, we still call for NGS and PD‐L1 testing in all patients with LFS to identify more potential therapeutic targets.

In the genetic analysis of *TP53*, its mutation sites are mostly distributed between exons 5 and 8, but there are 13.6% mutation sites outside this region (Soussi and Beroud 2001). The Case 4 we included was a patient with a mutation in *TP53* exon 4, and it was the only patient without distant metastasis among all patients. He suffered from two types of malignant tumors. When the first tumor appeared, he was only 33 years old. The occurrence time of these two tumors differed by more than a decade. On a recent inspection, there were no signs of tumor recurrence and/or metastasis. It follows that LFS is a cancer‐prone disease, but there may be a relatively inert class of molecules involved in the mutation, like R110L exon 4. Therefore, examining the entire coding region between exons 1–10 of the *TP53* gene, as well as introns that flank these exons, may help clinicians evaluate the prognosis of patients with LFS.

Currently, the management of asymptomatic *TP53* mutation carriers focuses on monitoring and treating high‐risk individuals and families at an early stage through clinical surveillance protocols (Villani et al. 2011; Leroy et al. 2017). This measure significantly improved the 5‐year overall survival of carriers of the germline *TP53* pathogenic variants (88.8% vs. 59.6%) (Villani et al. 2016). In the era of personalized and precision medicine, there is a growing need to identify hereditary predisposition syndromes, as it will allow appropriate genetic evaluation and facilitate timely screening, surveillance, and therapy for patients and their relatives.

## 5 Conclusion

In summary, LFS is characterized by genetic susceptibility to tumors. It usually occurs at a younger age and may present with two or more different types of malignant tumors. However, factors such as the different forms of germline *TP53* mutations and the timing of disease discovery often predict completely different prognoses. Therefore, it is necessary to adopt early detection and early treatment measures for members of LFS families. In addition, this new treatment model of targeted therapy and immunotherapy may improve the prognosis of patients with LFS to a certain extent.

## Author Contributions

All authors contributed to the study conception and design. Conceptualization was proposed by Meiyu Fang, Jun Cao, and Binbin Song; material preparation, data collection, and analysis were performed by Keyu Chen and Yuyang Gu; the first draft of the manuscript was written by Keyu Chen and Xiaofang Xu; review and editing were finished by Jun Cao and Yufen Xu; supervision was executed by Meiyu Fang, Jun Cao, and Binbin Song. All authors commented on previous versions of the manuscript. All authors read and approved the final manuscript.

## Funding

This study was supported by National Natural Science Foundation of China (81702653 to J.C.).

## Ethics Statement

This study involving human participants was in accordance with the ethical standards of the institutional and national research committee and with the 1964 Helsinki Declaration and its later amendments or comparable ethical standards. The Human Investigation Committee (IRB) of Zhejiang Cancer Hospital approved this study.

## Conflicts of Interest

The authors declare no conflicts of interest.

## Data Availability

The datasets involved in this study will not be made public due to containing patients' privacy, but are available from the corresponding author on reasonable request.
